# Co‐Producing an Empathy‐Focused Medical Curriculum With Patients, Educators, and Students

**DOI:** 10.1111/tct.70100

**Published:** 2025-05-07

**Authors:** Amber Bennett‐Weston, Cathy Harrell, Andy Ward, Max Jones, Jeremy Howick

**Affiliations:** ^1^ Stoneygate Centre for Empathic Healthcare, Leicester Medical School University of Leicester, George Davies Centre Leicester UK; ^2^ Leicester Medical School University of Leicester, George Davies Centre Leicester UK

**Keywords:** co‐production, curriculum development, medical education, patient involvement, undergraduate

## Abstract

**Background:**

Medical curriculum development rarely involves patients, educators, and students, meaning that key stakeholders' voices are not adequately represented in curricular content. In this paper, we describe the co‐production of an empathy‐focused medical curriculum involving patients, educators, and students.

**Approach:**

We adopted the National Institute for Health Research co‐production principles to develop three curriculum streams: 1) delivering evidence‐based empathy lectures, 2) involving patients in biomedical science teaching, and 3) implementing longitudinal empathic communication and clinical skills teaching. Patients, educators, and students were purposefully sampled from one medical school. At least one co‐production workshop was conducted for each curriculum stream, combined with written engagement. All participants were prepared, and patients were remunerated.

**Evaluation:**

Four co‐production workshops took place between July 2023 and April 2024. There were 24 participants (eight patients, eight educators, and eight students). Co‐production developed new lectures on the importance of therapeutic empathy, meaningful approaches to active patient involvement in biomedical science lectures, and communication and clinical skills training re‐framed from a patient perspective. A longitudinal evaluation will assess the impact of these changes on students' empathy.

**Implications:**

Co‐producing a medical curriculum can lead to important curricular changes, including greater integration of the patient voice across a five‐year programme. Five key learning points can assist curriculum developers in their future efforts to co‐produce medical curricula: 1) appoint an independent point‐of‐contact to support participants, 2) create a feedback loop, 3) encourage various modes of engagement, 4) explore and be open to all contributions, and 5) be transparent about the process.

## 1 Background

Therapeutic empathy (sometimes called “clinical empathy”) involves healthcare practitioners exploring and understanding patients' perspectives, engaging emotionally, and taking therapeutic action while maintaining professional boundaries [1]. In practice, therapeutic empathy improves outcomes for patients (reduced pain and mortality, and improved satisfaction) [2] and practitioners (reduced burnout and improved job satisfaction) [3]. Conversely, a lack of therapeutic empathy can increase medical errors [4].

Despite its benefits [2, 3], medical students' empathy appears to decline throughout their training [5]. This has been attributed to a hidden curriculum that includes a stressful workload, an over‐emphasis on biomedical knowledge, and un‐empathic role‐models [6]. Developing curriculum‐wide interventions that help students to overcome the problematic hidden curriculum by making connections between biomedical knowledge and patients' perspectives, and identifying empathic role‐models, has been identified as a priority for medical schools [6].

Medical curriculum development remains largely in the hands of medical educators [7, 8]. Although patients (by which we mean people with health conditions and their carers) [9] are increasingly actively involved in education [10], they are rarely involved in curriculum development [8]. Students are similarly seldom included in this process [7]. There are even fewer examples of patients, educators, and students working together to develop medical curricula [7, 8]. This is despite expectations for medical schools globally to adopt a paradigm of social accountability [9] and, in the United Kingdom (UK), the General Medical Council's (GMC) requirement that medical curriculum development “be informed by medical students … educators … and patients…” [11] As key stakeholders in medical education, students (as the recipients of curricula) and patients (as recipients of the care provided by those students) should have a voice in shaping medical curricula [12].

*Medical curriculum development remains largely in the hands of medical educators.… As key stakeholders in medical education, students … and patients … should have a voice in shaping medical curricula*.

Co‐production can address this gap by bringing patients, educators, and students together to develop medical curricula through dynamic discussions that value diverse perspectives [13, 14]. Co‐production refers to stakeholders working together, “sharing power and responsibility from the start to the end of a project…” [15]. In healthcare research, the concept of co‐production is well‐established [15]; however, little is known about how the principles of co‐production can be applied to medical curriculum development [12]. In this paper, co‐authored with a patient and a student, we describe our approach to co‐producing an empathy‐focused medical curriculum with patients, educators, and students.

## 2 Approach

### 2.1 Design

In the absence of best‐practice guidance for co‐production in curriculum development, we adopted the National Institute for Health and Care Research (NIHR) guidance on co‐production in research [15]. This guidance sets out five key principles [15]. Table 1 summarises how we applied these principles in our work.

**TABLE 1:** Implementation of the NIHR's [15] key co‐production principles to develop an empathy‐focused medical curriculum.

Key principle of co‐production	Description	Approach
**Sharing of power**	The project is jointly owned and people work together to achieve joint understanding.	Neither a patient, educator, nor student led the workshops. Researchers facilitated the workshops to ensure equal participation across stakeholder groups. Participants were divided into mixed breakout groups to promote collaboration and prevent any single group from dominating. The workshops were held in a neutral, familiar location (Leicester Medical School meeting room) to minimise power dynamics.
**Including all perspectives and skills**	The research team includes all those who can make a contribution.	Participants included patients, educators, and students. Meetings were held in accessible locations with accessible documentation (large font, plain English). Multiple engagement methods were offered: in‐person workshops, email, and online collaborative documents.
**Respecting and valuing knowledge of all involved**	Everyone is of equal importance.	An ice‐breaker had participants reflect on each stakeholder's unique value. The group created ground rules about respecting all views. Leicester Medical School has an already established commitment to patient involvement, which helped to ensure equal value was placed on lived experience, pedagogical expertise, and medical knowledge.
**Reciprocity**	Everybody benefits from working together.	All participants benefitted from the co‐production process. Patients were remunerated for their time, and developed their skills in curriculum development that could be applied and further developed in future involvement opportunities. Students acquired experience that would support future job applications. Educators had the opportunity to improve their existing teaching. Several participants took on roles in the delivery of the teaching developed through the co‐production workshops.
**Building and maintaining relationships**	An emphasis on relationships is key to sharing power.	Regular communication was maintained throughout the co‐production process and has continued during the evaluation of the curriculum streams. This was essential to ensure participants understood how their input had made a difference to the curriculum and students' empathy.

### 2.2 Context

The Stoneygate Centre for Empathic Healthcare launched in 2023 and is based at Leicester Medical School (LMS). The Centre's remit is to embed empathy into the 5‐year medical curriculum, involving a pre‐clinical phase (years 1 and 2) and a clinical phase (years 3, 4, and 5). We sought to co‐produce three evidence‐based [6] curriculum streams: 1) delivering evidence‐based empathy lectures, 2) involving patients in biomedical science teaching, and 3) implementing longitudinal empathic communication and clinical skills teaching. Although the broad topic of each curriculum stream was evidence‐based [6], the learning outcomes, curricular content, and methods of teaching were not pre‐specified. We informed participants that the streams were based on prior research [6], but that their final form would be determined by the co‐production process.

### 2.3 Sampling and Recruitment

We purposefully sampled a minimum of two patients, two educators, and two students for each workshop. A mix of clinical and non‐clinical educators with different areas of expertise, and students from each medical school year, were recruited from over 200 educators and 1400 students at LMS. Patients with different lived experiences of health and care were recruited from LMS's Patient and Carer Group. This group is comprised of approximately 140 members who are recruited by practitioners, local charities, and word‐of‐mouth. Remuneration was offered to all patients. A point‐of‐contact was recruited to support participants.

### 2.4 Innovation

We conducted at least one co‐production workshop for each curriculum stream. All participants were provided with a preparatory document describing the background to, and aims of, the co‐production work, along with the definition of therapeutic empathy outlined in the opening sentence of this paper [1]. Participants had the opportunity to ask questions before the workshops, or to have a one‐to‐one meeting with the point‐of‐contact. Each workshop lasted for one and a half hours and was facilitated by two researchers from the Centre. The workshops aimed to develop learning outcomes and methods of teaching for each curriculum stream, following a generic structure (Table 2).

**TABLE 2:** Structure of the co‐production workshops.

Activity	Description	Time allotted
**Welcome and introductions.**	Ice‐breaker exercise: facilitators and participants were asked to identify one personal or professional quality or experience that they think added value to the co‐production process. Then, as a group, agree ground rules to underpin the workshop(s).	15 min
**Aims of the workshop.**	Facilitators describe the aims of the day and how these will be achieved, and provide opportunities for participants to ask questions.	5 min
**Breakout groups: learning outcomes.**	Small groups of at least one patient, one educator, and one student were assembled. A facilitator was assigned to each small group. Working with the relevant curriculum stream proposal, participants were asked to identify what they wanted students to take away from the stream.	20 min
**Breakout groups: teaching methods.**	Once they had compiled a list of take away points, the small groups were asked how they thought these could be achieved. Questions were used as prompts, for example: what experiences did the students need to have in order to learn? What did the educator need to do to support this learning? How could a teaching session be structured to facilitate learning?	20 min
**Feedback and action planning.**	Each small group provided feedback on their discussions to the whole group. Sequentially, they discussed learning outcomes and teaching methods for the curriculum stream. The group then discussed similarities and differences between their suggestions, and voted for the best approach for that particular curriculum stream. The approach with the majority vote was taken forward. This was written up by one facilitator.	30 min

After each workshop, one author sent a summary of the proposed curriculum stream plan to all participants. Participants were encouraged to contribute to an online, collaborative version of this document via Google Docs. Once all participants confirmed they were happy with the curriculum stream, a poll was circulated to ascertain whether further workshops were needed. This process continued iteratively until participants confirmed they were satisfied with the curriculum stream.

### 2.5 Ethics Statement

Ethical approval was obtained from the University of Leicester's Medicine and Biological Sciences Research Ethics Committee (ref: 40270‐aw139‐ls:medicine).

## 3 Evaluation

Four co‐production workshops took place between July 2023 and April 2024. One workshop was conducted for curriculum stream 1, another for curriculum stream 2, and two workshops were conducted for curriculum stream 3. A total of 24 participants (eight patients, eight educators, and eight students) were involved (Table 3). Patients represented lived experiences of long‐term conditions, disability, cancers, stroke, maternity, and mental illness, and all had prior experience of being involved in health professions education (years of involvement ranging from 3 to 10), including sharing their lived experiences in small‐group teaching, contributing to curriculum development, or supporting student selection. Educators were involved in teaching across the pre‐clinical and clinical curriculum phases and represented diverse expertise including in communication skills, gastroenterology, and reproduction. Students participated from each year of the medical degree.

**TABLE 3:** Participant characteristics by co‐production workshop.

Co‐production workshop	Participant	Gender	Ethnicity
**Delivering evidence‐based empathy lectures**	Patient 1	Female	Black, Black British, Caribbean, or African
	Patient 2	Male	White
	Educator 1	Male	Asian or Asian British
	Educator 2	Male	Asian or Asian British
	Student 1	Male	Black, Black British, Caribbean, or African
	Student 2	Female	White
**Involving patients in biomedical science teaching**	Patient 3	Female	White
	Patient 4	Female	White
	Educator 3	Male	White
	Educator 4	Female	White
	Educator 5	Female	White
	Student 3	Female	White
	Student 4	Female	White
**Implementing longitudinal empathic communication and clinical skills teaching (workshop 1)**	Patient 5	Male	White
	Patient 6	Female	Asian or Asian British
	Educator 6	Male	White
	Educator 7	Male	Asian or Asian British
	Student 5	Female	White
	Student 6	Male	White
**Implementing longitudinal empathic communication and clinical skills teaching (workshop 2)**	Patient 7	Female	White
	Patient 8	Female	White
	Educator 8	Female	White
	Student 7	Male	White
	Student 8	Male	Asian or Asian British

### 3.1 Implementation

Several curricular changes were proposed, including new sessions on therapeutic empathy (across years 1 to 5), meaningful and innovative approaches to involving the patient voice in biomedical science teaching (in years 1 and 2), and communication and clinical skills teaching that had been re‐framed through an empathic lens (across years 1 to 5) [6, 16].

Reaching an agreement on each curricular change sometimes involved disagreements. For example, while educators were concerned around tokenistic patient involvement in biomedical science lectures, patients thought that this could be mitigated through adequate preparation and remuneration, and students agreed that it would enhance learning. As such, the combination of—and sometimes conflict between—different perspectives enriched the co‐production process.

*… the combination of – and sometimes conflict between – different perspectives enriched the co‐production process*.

The first‐step in evaluating the co‐production process involved mapping the implementation of the proposed curricular changes. We considered this important because a key goal of co‐production is to bring about meaningful change to established practices [14]. Implementation of the curricular changes is being progressed at different stages; curriculum streams 1 and 2 have been fully implemented; implementation of stream 3 is ongoing (Table 4).

**TABLE 4:** Curricular changes based on the outcomes of the co‐production process and their implementation status.

Curriculum stream	Brief proposal for curriculum stream	Curricular change(s) based on co‐production outcomes	Implementation status
**Delivering evidence‐based empathy lectures**	Deliver one‐hour empathy lectures about what therapeutic empathy is, what students can expect from the empathy‐focused curriculum and why empathy is important.	1. The empathy lectures are new teaching sessions that are delivered at the beginning of each academic year to students in every year of the medical degree. 2. The learning outcome for the lectures is to be able to describe what therapeutic empathy is, its importance for patients and practitioners, and how it will be taught. 3. The lectures are delivered jointly by patients, educators, and students. Each stakeholder will be given 10 min to deliver their part of the lecture: A patient will begin by sharing their experiences of empathy (positive or negative), and its impact. An educator will define therapeutic empathy, outline the evidence‐based, and describe what to expect from each year of the empathy focused curriculum. A student will reflect on their experience of being on placement and observing or delivering empathic care. 4. Student engagement is encouraged by punctuating each 10‐min part of the lecture with questions for them to answer anonymously and digitally. For example: what does empathy mean to you?	Fully implemented
**Involving patients in biomedical science teaching**	Involve patients in lecture‐based biomedical science teaching during the pre‐clinical phase of training (years one and two).	1. Patients will be involved in existing biomedical science lectures for medical students in the pre‐clinical phase of training. 2. The learning outcomes for the lectures are: To link theory to practice (and patient presentations); To recognise the psychosocial impact of disability and/or disease. 3. Patients are invited to share their lived experiences of a particular health condition for 10–15 min at the end of a biomedical science on that condition or bodily system, followed by a short Q&A session with students. 4. Patients are prepared by an educator who provides an overview of the lecture, and how their experience contributes. Together, they agree what they are comfortable sharing and answering questions about. A debrief is provided after the lecture for patients to address any concerns. 5. Remuneration is offered for patients' preparation for, and contribution to, the lecture.	Fully implemented
**Implementing longitudinal empathic communication and clinical skills teaching**	Enhance the empathy focus of communication and clinical skills teaching throughout the medical degree (years one to five).	1. Existing teaching on communication and clinical skills has been, or will be, modified. 2. A programme titled “Understanding Patients” is being implemented into the pre‐clinical phase of training to provide students with a longitudinal attachment to a patient. Students will visit patients in their homes to vicariously experience what it is like to be a patient. 3. Regular formative, workplace‐based assessments focused on students' interactions with patients to be embedded into the clinical phase of training across diverse clinical specialities such as surgery and emergency medicine, rather than limiting it to areas like palliative care. 4. Students in the clinical phase of training to be required to collect a specified number of patient‐reported assessments of their empathy levels in order to successfully complete clinical placements.	Implementation ongoing

### 3.2 Improvement

The second step in the evaluation involves measuring improvements to students' empathy, student satisfaction, and experiences of the empathy‐focused curriculum, following implementation of the curricular changes. This stage of the evaluation is ongoing, involving a 5‐year longitudinal study described elsewhere [16]. Each medical school year, students' empathy levels are measured using self‐reported and patient‐reported empathy scales [16]. Routine student surveys capture satisfaction, and interviews with small numbers of students from each year‐group explore their experiences of the curriculum [16].

**FIGURE 1:**
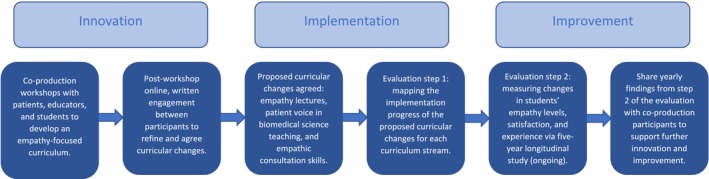
Stages of innovation, implementation, and improvement for co‐producing an empathy‐focused medical curriculum.

The findings from each year of this longitudinal study will be shared with the co‐production workshop participants, providing opportunities for further innovation of the curriculum and, subsequently, further improvement. In this way, the process of co‐production is iterative, ongoing, and embedded within our curriculum. Figure 1 summarises the stages of innovation, implementation, and improvement for the co‐production process.

## 4 Implications

Co‐production with patients, educators, and students led to important changes to medical curricula to improve students' empathy [5]. This is important in light of the current dearth of evidence of patient *and* student involvement in medical curriculum development [7, 8].

*Co‐production with patients, educators, and students led to important changes to medical curricula to improve students' empathy*.

The under‐representation of the patient voice in medical curriculum development has been attributed to the fear that it will come at the expense of existing curricular content [12]. Our findings partly address this issue, showing that involving patients in curriculum development allows existing teaching to be reframed from a patient perspective. This is likely to better equip students to understand and meet their patients' needs, supporting the social accountability of medical curricula [9, 12]. In this way, our work highlights the added value of involving patients in curriculum development and underscores the importance of seeing patients' lived experience and practitioners' medical knowledge as complementary, not competitive.

*… our work highlights the added value of involving patients in curriculum development …*

Another barrier to involving stakeholders in curriculum development is the lack of guidance on *how* to do it [12]. One challenge we encountered was navigating power dynamics, particularly empowering participants to develop ideas and make decisions with minimal input from us. Participants often asked for our opinions on the feasibility and relevance of their proposed curricular changes. As such, we did influence the co‐production process by providing practical advice regarding what was achievable with the resources we had, but endeavoured for any contribution to be participant‐driven.

Based on our experiences, we have identified five key learning points to support curriculum developers in co‐producing medical curricula:

1. Appoint an independent point‐of‐contact to support participants throughout co‐production (particularly patients who may be less familiar with institutional policies and processes).
2. Create a feedback loop to communicate how participants’ input has been implemented and the difference it has made.
3. Encourage participants to engage in various ways to ensure all voices are heard (for example, in workshops, via email, and through written contributions).
4. Explore and be open to *all* contributions, and reflect, together, on if and how participants’ suggestions might be actioned.
5. Be transparent with participants about the stage of the work they are joining, where this sits within the curriculum, and how long they are likely to be involved.

### 4.1 Limitations

A limitation of this work is that despite our efforts to purposefully sample diverse participants, most were of White ethnicity, and all were based at one medical school in the UK. However, the application of co‐production principles and description of our context supports reflection about the transferability of our approach to other institutions. Another limitation is that the second step of the evaluation is ongoing [16]. Moreover, our evaluation did not involve collecting the workshop participants' experiences of the co‐production process. As such, future research should explore how key stakeholders perceive and experience co‐production, to inform best‐practice [12].

## 5 Conclusion

In conclusion, we have shown that patients, educators, and students can co‐produce and significantly influence an empathy‐focused medical curriculum. Bringing key stakeholders together has led to significant curricular changes that provide students with opportunities to develop therapeutic empathy through engagement with the evidence‐base, exposure to the patient voice, and vicarious experience of the patient perspective. Curriculum developers can use our approach to ensure that key stakeholders' voices are represented within medical curricula, thus helping to meet regulatory requirements [11].

## Author Contributions

**Amber Bennett‐Weston:** conceptualization, writing – original draft, methodology, writing – review and editing. **Cathy Harrell:** methodology, writing – original draft, writing – review and editing. **Andy Ward:** methodology, writing – original draft, writing – review and editing. **Max Jones:** writing – original draft, writing – review and editing. **Jeremy Howick:** conceptualization, methodology, writing – original draft, writing – review and editing.

## Conflicts of Interest

The authors declare no conflicts of interest.

## Data Availability

The data that support the findings of this study are available from the corresponding author upon reasonable request.
